# Adherence to healthful eating patterns and the risk of insomnia in postmenopausal women: insights from the Women’s Health Initiative study

**DOI:** 10.1093/sleep/zsaf365

**Published:** 2025-11-12

**Authors:** Emily J Arentson-Lantz

**Affiliations:** Department of Nutrition Sciences and Health Behavior, University of Texas Medical Branch, Galveston, TX, United States

Insomnia symptoms affect up to 60 per cent of peri- and postmenopausal women [1]. Persistent insomnia, particularly short sleep duration, is associated with negative health outcomes, notably cardiovascular disease risk which also increases in women following the menopause transition [2]. Identifying healthy lifestyle behaviors that attenuate the risk or persistence of insomnia among postmenopausal women is greatly desirable. Emerging evidence indicates individual food items and dietary patterns containing elevated levels of neuromodulating bioactives, including melatonin, fiber, tryptophan, and polyphenols, may support better sleep outcomes [3]. However, much of this data is cross-sectional or short-term in nature. What is often missing from the literature is a longitudinal perspective on the role of diet on poor sleep quality/insomnia. With 1.3 million women entering menopause every year in the United States, using observational data from existing longitudinal, robust cohorts are valuable to assess the impact of healthful eating patterns on insomnia risk over time to provide evidence-based diet recommendations for women seeking to lower their risk for insomnia.

In this work by Zuraikat and colleagues [4], they present an analysis of participants in the Women’s Health Initiative—Observational Study (WHI-OS; postmenopausal women with mean age 63 years) that evaluated how adherence to established healthful eating patterns were prospectively and longitudinally associated with the insomnia incidence. The healthful eating patterns included either the alternative Mediterranean diet (aMed) or the Dietary Approaches to Stop Hypertension (DASH) diet; both dietary patterns are characterized by consumption of fruits, vegetables, nuts, and legumes, which have been previously linked to improved sleep outcomes. Women were grouped based on presence or absence of insomnia symptoms at baseline and 3 years as (i) *stable or new onset insomnia* (defined as insomnia at baseline and 3 years/no insomnia at baseline, but yes at 3 years) or (ii) s*table absence or remission of insomnia* (no insomnia at either baseline or 3 years/insomnia at baseline but no at 3 years). Among the WHI-OS cohort, 30.2 per cent of participants reported insomnia symptoms at baseline (*n* = 22 551), with 9349 new cases of insomnia reported at the three-year follow up. Women who reported no insomnia at baseline (50 644 participants) with good adherence to the aMed and DASH had 7.5 per cent (CI: 0.879–0.974) and 6.3 per cent (CI: 0.891–0.985) lower odds, respectively, of developing insomnia at Year 3 follow-up (compared to those with poor adherence) each standard deviation increase in aMed and DASH adherence was associated with 3.8 per cent and 4.1 per cent lower risk for risk of insomnia at 3 years. Longitudinal assessment of 74 513 participants indicated that women with good diet quality at baseline based on aMed or DASH scoring criteria had 6.3 per cent (0.903–0.971) to 8.5 per cent (0.883–0.948), respectively, lower odds of having stable or new onset insomnia between baseline and Year 3.

While the authors employed rigorous statistical models that controlled for numerous demographic and health variables assessed by the WHI-OS, the observational design, and the use of a single food frequency questionnaire to assess dietary pattern adherence present limitations in establishing a causative relationship between diet and sleep. The participants in the WHI-OS were largely white, non-Hispanic women, and well-educated with >70 per cent having at least some college education which also limits generalizability. Socioeconomic status and minority race and ethnicity are risk factors for poor sleep/insomnia; thus, future randomized clinical trials to evaluate interventions to increased adherence to healthful dietary patterns should include a more diverse population. Since good adherence to the dietary patterns was defined by the scores of the highest two quintiles of the dataset, it is not clear if the dietary quality of this population was relatively higher than the general population.

An important discussion point highlighted by Zuraikat et al. [4] is that the while the 6–8 per cent reduction in insomnia onset or remission in insomnia symptoms may seem modest, it is comparable to the magnitude in reduction of cardiovascular disease risk associated with greater diet quality. However, an important consideration in translating the findings from this study is the age of the dataset. The WHI-OS was conducted nearly 30 years ago. Since then, a number of factors have emerged that may shape the risk for insomnia and/or poor sleep quality, including the pace of a 24/7 lifestyle amplified by the usage of smartphones with incessant dings and alerts, life stressors (e.g. COVID-19), and the rise in obesity and chronic disease prevalence. It is unclear if adherence to a dietary pattern would have the same magnitude of impact in the insomnia symptoms in a contemporary cohort of postmenopausal women. Though it can be argued that following a healthful dietary eating pattern is generally associated with other health benefits (lower risk of obesity, hypertension, type II diabetes), which may contribute to a lower risk for insomnia.

A growing number studies like that from Zuraikat et al. [4] generate convincing evidence of a relationship between diet quality and sleep. Looking forward to the future, there are several priorities for planning future clinical trials to refine and test the relationship between diet and sleep. Firstly, is that when feasible, collecting and reporting descriptive data on diet quality when conducting studies where sleep is an outcome of interest. It is the customary for physical activity interventions studies to collect and report dietary data because dietary quality/dietary components impact outcomes of interest (and vice versa)—sleep intervention studies may consider adding in this outcome measure as well. Secondly in population of postmenopausal women, the consumption of nuts and legumes was associated with a decrease in incident risk of insomnia among participants with good adherence to the DASH diet. While nuts and legumes are postulated to improve sleep because of increased melatonin content, lower glycemic index, higher fiber content, etc., there are relatively few studies that have prospectively tested any individual items from this food group or studied this food group as a whole in randomized controlled trials. Future work should consider if the impact of nuts and legumes on insomnia risk.

Findings from this work from Zuraikat et al. [4] lay the groundwork tailoring much-need messaging for postmenopausal women on the importance of a healthful diet in mitigating insomnia risk.
